# Integrative metagenomic and lipidomic analyses reveal alterations in children with obesity and after lifestyle intervention

**DOI:** 10.3389/fnut.2024.1423724

**Published:** 2024-09-10

**Authors:** Chunyan Yin, Lujie Liu, Dong Xu, Meng Li, Min Li, Yujie Qin, Bei Zhang, Yongfa Sun, Yuesheng Liu, Yanfeng Xiao

**Affiliations:** ^1^Department of Pediatrics, The Second Affiliated Hospital of Xi’an Jiaotong University, Xi’an, China; ^2^Department of Pediatrics, Tongji Medical College, Tongji Hospital, Huazhong University of Science and Technology, Wuhan, China; ^3^Department of Pediatrics, Luoyang Central Hospital, Luoyang, China

**Keywords:** childhood, obesity, metagenomics, lipidomic, weight loss

## Abstract

**Background:**

Despite emerging evidence linking alterations in gut microbiota to childhood obesity, the metabolic mechanisms linking gut microbiota to the lipid profile during childhood obesity and weight loss remain poorly understood.

**Methodology:**

In this study, children with obesity were treated with lifestyle weight loss therapy. Metagenomics association studies and serum untargeted lipidomics analyses were performed in children with obesity and healthy controls before and after weight loss.

**Main findings:**

We identified alterations in gut microbiota associated with childhood obesity, as well as variations in circulating metabolite concentrations. Children with obesity showed significant decreases in the levels of *s-Rothia_kristinae* and *s-Enterobacter_roggenkampii*, alongsige elevated levels of *s-Clostridiales_bacterium_Marseille-P5551*. Following weight loss, the levels of *s-Streptococcus_infantarius* and *s-Leuconostoc_citreum* increased by factors of 3.354 and 1.505, respectively, in comparison to their pre-weight loss levels. Correlation analyses indicated a significant positive relationship between ChE(2:0) levels and both with *s-Lachnospiraceae_bacterium_TF09-5* and fasting glucose levels. CoQ8 levels were significantly negatively correlated with *s-Rothia_kristinae* and HOMA-IR.

**Conclusion:**

We linked altered gut microbiota and serum lipid levels in children with obesity to clinical indicators, indicating a potential impact on glucose metabolism via lipids. This study contributes to understanding the mechanistic relationship between altered gut microbiota and childhood obesity and weight loss, suggesting gut microbiome as a promising target for intervention.

**Clinical trial registration:**

https://www.chictr.org.cn/showproj.html?proj=178971, ChiCTR2300072179.

## 1 Introduction

The prevalence of childhood obesity is reaching unprecedented levels and is estimated to affect 159 million children worldwide (1). Metabolic disorders caused by obesity affect the normal growth and development of children, as well as increase the risk of type 2 diabetes mellitus, metabolic syndrome, and cancer (2). The management of childhood obesity primarily relies on lifestyle interventions, encompassing dietary modifications, physical activity, and lifestyle adjustments. However, the adherence to and efficacy of these interventions have been suboptimal. Furthermore, the unique characteristics of the pediatric population pose challenges to the implementation of pharmacological treatments and bariatric surgery (3). Therefore, it is imperative to identify effective and feasible targets for the treatment and prevention of childhood obesity.

In recent years, a growing body of research has demonstrated that gut microbiota is the primary endogenous factor affecting obesity (4). Owing to the substantial variation in the composition of gut microbiota among individuals, as well as variations in gene expression and functions within the same individual, elucidating the relationship between gut microbiota and childhood obesity, as well as its mechanism of action, poses a challenge. The abundance, diversity, and stability of human gut microbiota have a significant correlation with the occurrence and development of childhood obesity. This can affect energy metabolism pathways in these children through various factors, such as intrauterine flora exposure, delivery mode, and geographical factors, thereby leading to intestinal flora disorder and promoting childhood obesity. Several studies have found that the gut microbiota of children and adults with obesity is characterized by an increase in Firmicutes and a decrease in Bacteroidetes abundance (5–9). At the genus level, increased levels of *Blautia*, *Eubacterium*, and *Bifidobacterium* and decreased levels of *Bacteroides*, *Ruminococcus*, and *Akkermansia* have been found in children with obesity compared with those in normal-weight children (10–12). Most of the aforementioned studies used 16 s RNA sequencing to detect gut microbiota. A few studies have examined gut microbiota and described functional alterations in children with obesity based on metagenomics (13). The composition and function of gut microbiota are dynamic. Lipids affect gut microbiota both by acting as substrates for bacterial metabolism and signaling to regulate bacterial growth (14). Investigating the relationship between gut microbiota and lipids may explain the pathogenesis of childhood obesity and provide new therapeutic targets for childhood obesity.

The primary etiology of obesity lies in the increased consumption of energy-dense foods rich in fat and sugar, coupled with a lack of physical activity. When the adipose tissue surpasses its maximal threshold for lipid storage, surplus lipids are released into the circulation and other organs, thereby inducing lipotoxicity (15). Untargeted lipidomics provides a new perspective for studying childhood obesity and its associated complications. Lau et al. (16) conducted a targeted metabolomics analysis on 1,162 European children and observed a negative correlation between body mass index (BMI) and lysophosphatidylcholine (LPC)(14:0) and LPC(16:1) levels, whereas long-chain LPCs showed a positive correlation with BMI. Furthermore, the effect of visceral adiposity on cardiovascular diseases in children with obesity was mediated by decreased phosphatidylcholine (PC)(16:0_2:0) and increased LPC(14:1_0:0) levels. Notably, the stimulation of endoplasmic reticulum stress by LPC leads to mitochondrial dysfunction and increased apoptosis (17). Lopez et al. (6) studied the roles of ceramide and adiponectin in 28 female adolescents aged 10–17 years. Compared with healthy subjects, girls with obesity showed higher C(18:0), C(20:0), and C(22:0) ceramide and C(24:1) dihydroceramide levels. In children with obesity, the plasma levels of glycerophosphatides are generally low, whereas PC(36:2), LPC(18:1), LPC(18:2), and LPC(20:4) levels increase after weight loss (18–20). In an isocaloric fructose restriction trial, total and subspecies ceramide levels decreased significantly after weight loss in children with obesity. Increased ceramide levels may cause mitochondrial dysfunction and lead to insulin resistance (21). However, studies on untargeted lipids in children are scarce (13). It is essential to obtain data on lipid changes before and after weight loss in children from different regions to comprehensively characterize the lipid profiles of children with obesity.

In the present study, we aimed to compare the gut microbiome and lipidomics of healthy children and children with obesity before and after weight loss. Additionally, we investigated whether altered gut microbiota modulated host metabolism via serum lipids in children with obesity by performing multi-omics data analysis.

## 2 Materials and methods

### 2.1 Study cohort

Children with obesity visiting the clinic of the Pediatric Endocrinology Department at the Second Affiliated Hospital of Xi’an Jiaotong University who sought anti-obesity treatment were consecutively enrolled. The participants and their parents underwent examinations before and after treatment and provided blood and stool samples. The study inclusion criteria for children with obesity were age 6–18 years and age-and sex-specific BMI ≥ 95th percentile. Patients were excluded for recent weight loss of ≥5%, infective disease, immunodeficiency, prior transplantation, type 1 diabetes mellitus, inborn errors of metabolism, endogenous obesity, drug-induced obesity, and mental health conditions. Healthy children who visited a child healthcare clinic during the same period were included as controls. All participants and their parents agreed to participate in the study and provided written informed consent. This study was registered with the Chinese Clinical Trial Registry (Registration number: ChiCTR2300072179). This study was approved by the Second Affiliated Hospital of Xi’an Jiaotong University (no. 2022245).

### 2.2 Study design

**Figure 1:**
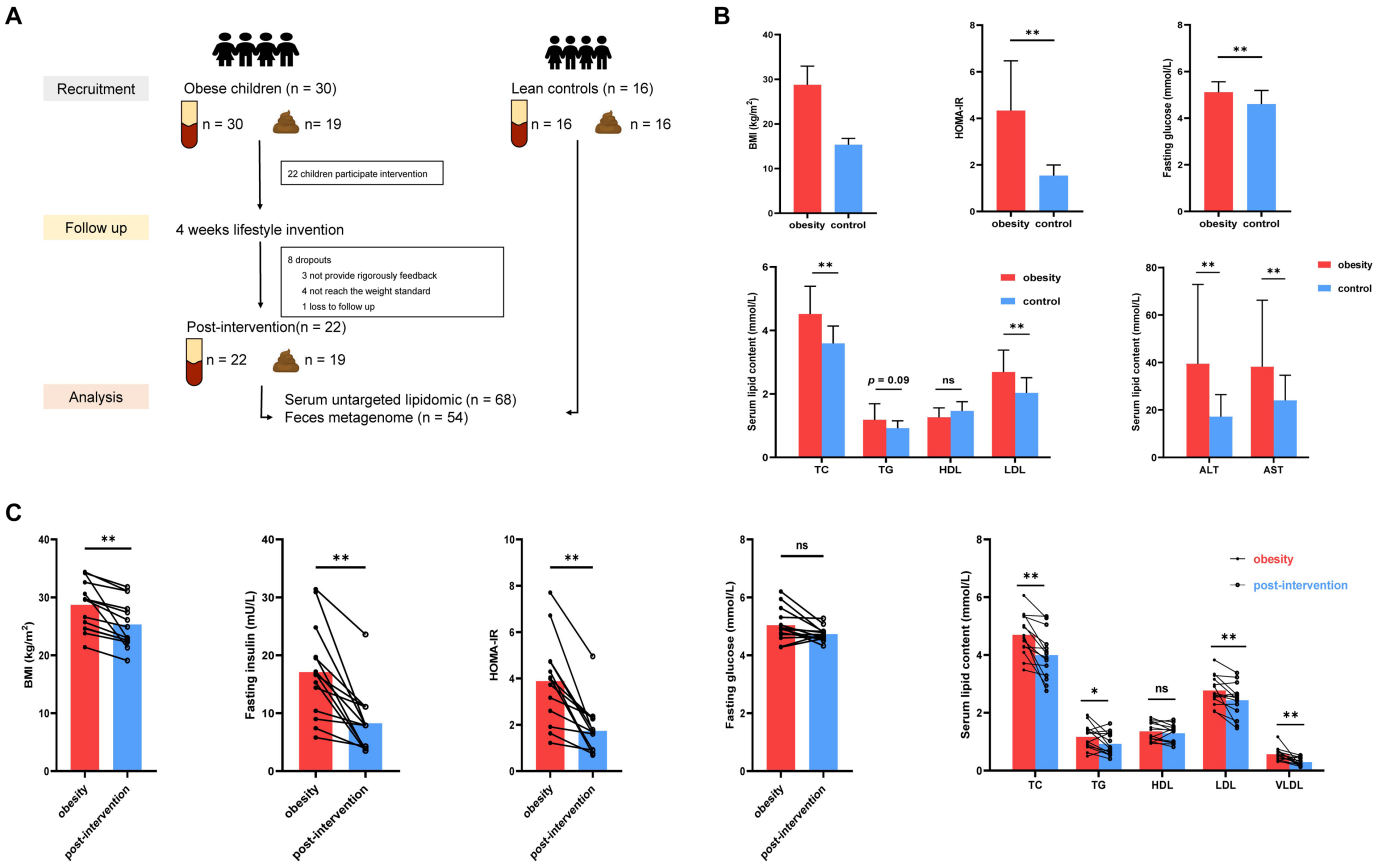
Alterations in clinical parameters in control and children with obesity at before and after weight loss intervention. **(A)** Workflow of clinical research in this study. **(B)** Comparison of BMI standard deviation score (BMI-SDS), fasting glucose, HOMA-IR, homeostatic model assessment of insulin resistance (HOMA-IR), alanine aminotransferase (ALT), aspartate aminotransferase (AST), triglyceride (TG), total cholesterol (TC), low-density lipoprotein (LDL) and high-density lipoprotein (HDL) between controls and children with obesity. **(C)** Comparison of BMI, fasting insulin, fasting glucose, HOMA-IT, TC, TG, HDL, LDL and very low-density lipoprotein (VLDL) in children with obesity at baseline and after weight loss. *p < 0.05, **p < 0.01.

Twenty-two children with obesity participated in a family-based lifestyle intervention program consisting of three main components. (i) Dietary intervention: First, after evaluating the nutritional composition and quality of food the children consumed in the last 3 days using nutrition calculation (Feiyang, Beijing), a trained dietitian developed an appropriate and strict diet for each child. Each parent received a separate diet sheet that listed the complete and diversified diet that could be replaced at each meal and was labeled with raw weight in grams. The daily oil intake was limited to 6 g. The final dietary recommendations were based on a balanced distribution of carbohydrates (50–70%), proteins (15–20%), and lipids (15–25%), to reduce daily intake by 350 kcal. (ii) Exercise program: The participants were required to exercise for 30 min every day, and the recommended sports included brisk walking, jogging, rope skipping, ball games, and swimming. In addition, sedentary activities and screen time were limited to <2 h per day. Parents of children with obesity sent daily diet photos to the researchers through mobile app group chat, recorded daily exercise through the exercise bracelet. Finally, the researchers collected follow-up data through the red-cap system. An overview of the study flow CONSORT diagram and the experimental protocol (Figure 1A) is provided.

### 2.3 Clinical and biochemical assessment

Standing height, body weight, and waist circumference were measured using a calibrated scale and stadiometer. BMI was calculated from height and weight. The BMI data were converted into a BMI-SDS according to the Chinese children’s and adolescents’ age-and sex-specific percentile standards (22). Blood samples were collected after overnight fasting, and the serum samples were stored at −80°C after centrifugation. For fecal sample collection, the parents and children were asked to follow a rigid standard: (i) wash their hands with a hand sanitizer, (ii) drain their urine, and (iii) collect stool samples into standard tubes. The collected stool specimens were quickly placed into liquid nitrogen cold raffinate and stored at −80°C until experiments.

Plasma glucose and lipid profiles (including TG, TC, HDL, LDL, VLDL, ALT, and AST) were tested using the Hitachi 747 autoanalyzer. Serum insulin levels were measured using radioimmunoassay. The HOMA-IR was used to estimate insulin resistance.

### 2.4 Serum metabolomics

#### 2.4.1 Metabolite extraction

An appropriate liquid sample, 800 μL of methyl tertiary butyl ether, and 240 μL of precooled methanol were added to a centrifuge tube and vortexed. The samples were sonicated, centrifuged, and dried under nitrogen gas. For mass spectrometry (MS) analysis, 200 μL of the 90% isopropanol/acetonitrile solution was added, redissolved, and centrifuged. The supernatant was collected for further analysis. The samples were separated using the UHPLC Nexera LC-30A ultra-high-performance LC system. Electrospray ionization positive and negative ion modes were used for MS detection. LipidSearch was used to perform peak identification, peak extraction, and lipid identification of lipid molecules and internal standards. The main parameters were a precursor tolerance of 5 ppm, a product tolerance of 5 ppm, and a product ion threshold of 5%. The Base Peak spectra of quality control (QC) samples was performed to examine the stability of the instrument. Principal component analysis (PCA) was used to check the repeatability of the experiment. The relative standard deviation (RSD) of ion peak abundance in QC samples was performed to identify the reliability of the data quality.

### 2.5 Fecal whole-genome shotgun sequencing

Total DNA was isolated from fecal samples using QIAamp Fast DNA Stool Mini Kit (Qiagen, United States). The library preparation protocol was performed and paired-end 2 × 150 bp sequencing was performed using the Illumina NovaSeq 6,000 high-throughput sequencing platform. Raw sequencing data were saved in the FASTQ format at: https://www.ncbi.nlm.nih.gov/bioproject/PRJNA1090975. The raw sequencing data were screened and filtered using fastp (v0.20.0) software. The effective sequences were annotated using Kraken2, and the bacterial, archaeal, fungal, protozoan, viral, metazoan, and green plant genome data from the RefSeq genome database of NCBI were used as references to construct a database with an adjusted confidence level of 0.5. A non-concatenated species abundance table was obtained after normalizing the species abundance. The MetaGeneMark software was used to analyze the contigs. The open reading frame was identified and the coding region was predicted to obtain the corresponding gene sequence, protein sequence, gene transfer format, and general feature format files. The filtered protein sequence sets were aligned with common protein databases to annotate gene functions in all samples.

### 2.6 Statistical analysis

Composition and abundance distribution tables were obtained for each sample at the six taxonomic levels. The α-diversity indices, rarefaction curve, species accumulation curve, rank abundance curve, and β-diversity were individually determined using QIIME software. Mothur software was used to calculate the abundance located in the top 50 of the Spearman rank correlation co-efficient between dominant species, for which |rho| > 0.6 and *p* < 0.01 were considered, and the related associated network of dominant species was constructed. The metagenomeSeq method was used to perform the pairwise comparisons of phylum-, genus-, and species-level taxa in the sample group, and the results of the statistical analysis of significant differences were obtained. The false discovery rate was controlled using the Benjamini–Hochberg method. Taxa with *adj.p* < 0.05 were selected as species with significant differences in abundance. KEGG, GO, EggNOG, and CAzmy annotations were performed based on protein annotation results and abundance. Enriched pathways between the groups were obtained by performing LEfSe analysis. Correlation analysis (Spearson’s correlation coefficients) between metabolomics, metagenomics, and clinical index was performed using the “corr.test” function.

Statistical analysis of clinical data was performed using SPSS 23.0 and GraphPad Prism 9.0 software. Normal distributions were assessed using the Kolmogorov–Smirnov test. Data are expressed as the mean ± standard error of the mean or count (percent). Comparisons between groups were performed using the *t*-test or chi-square test. Associations between clinical and metabolic variables were studied using Pearson’s or Spearman’s correlations. A *p* < 0.05 was considered statistically significant. Data were imported into Cytoscape and R software for visual display.

## 3 Results

### 3.1 Demographic and clinical data

Thirty children with obesity participated in this study. After 4 weeks, 22 children successfully lost more than 0.5% of their body weight. Blood and stool specimens were collected from children with obesity at baseline and after weight loss. Additionally, 16 age-matched children were recruited as healthy controls, and their blood stool samples were collected simultaneously. Eight children with obesity dropped out during the weight loss therapy because they did not strictly follow the protocol, did not meet the weight loss criteria, and were lost to follow-up. No significant differences were observed in the demographic characteristics according to sex or age between the obesity and control groups (Table 1). The obesity group showed significantly higher BMI standard deviation score (BMI-SDS), fasting glucose, homeostatic model assessment of insulin resistance (HOMA-IR), alanine aminotransferase (ALT), aspartate aminotransferase (AST), total cholesterol (TC), and low-density lipoprotein (LDL) levels than did the control group, whereas high-density lipoprotein (HDL) and triglyceride (TG) levels were similar in the two groups (*t*-test, Figure 1B). BMI decreased significantly after the intervention. Insulin and HOMA-IR levels significantly decreased, whereas no significant changes in fasting glucose levels were observed. Additionally, lipid metabolism was improved significantly. Serum TC, LDL, and very low-density lipoprotein (VLDL) levels decreased significantly after the intervention (paired *t*-test, Figure 1C).

**Table 1:** Clinical parameters of the study population.

Characteristics	Control (*n* = 16)	Obesity (*n* = 30)	Post-intervention (*n* = 22)
Gender male	8	24	16
Female	8	6	6
Age (years)	10.57 ± 2.77	10.79 ± 1.86	10.88 ± 1.81
Height	128.37 ± 17.29	151.09 ± 13.58	149.45 ± 10.05
Weight	25.86 ± 7.46	65.87 ± 16.73^a^	56.28 ± 15.05^a,b^
BMI (kg/m^2^)	15.32 ± 1.41	28.74 ± 4.18^a^	24.76 ± 4.16^a,b^
BMI-SDS	−0.30 ± 0.63	4.27 ± 1.80^a^	2.64 ± 1.37^a,b^

^ap < 0.05, compared with control. bp < 0.05, compared with the obesity group.^

### 3.2 Fecal metagenome analyses

**Figure 2:**
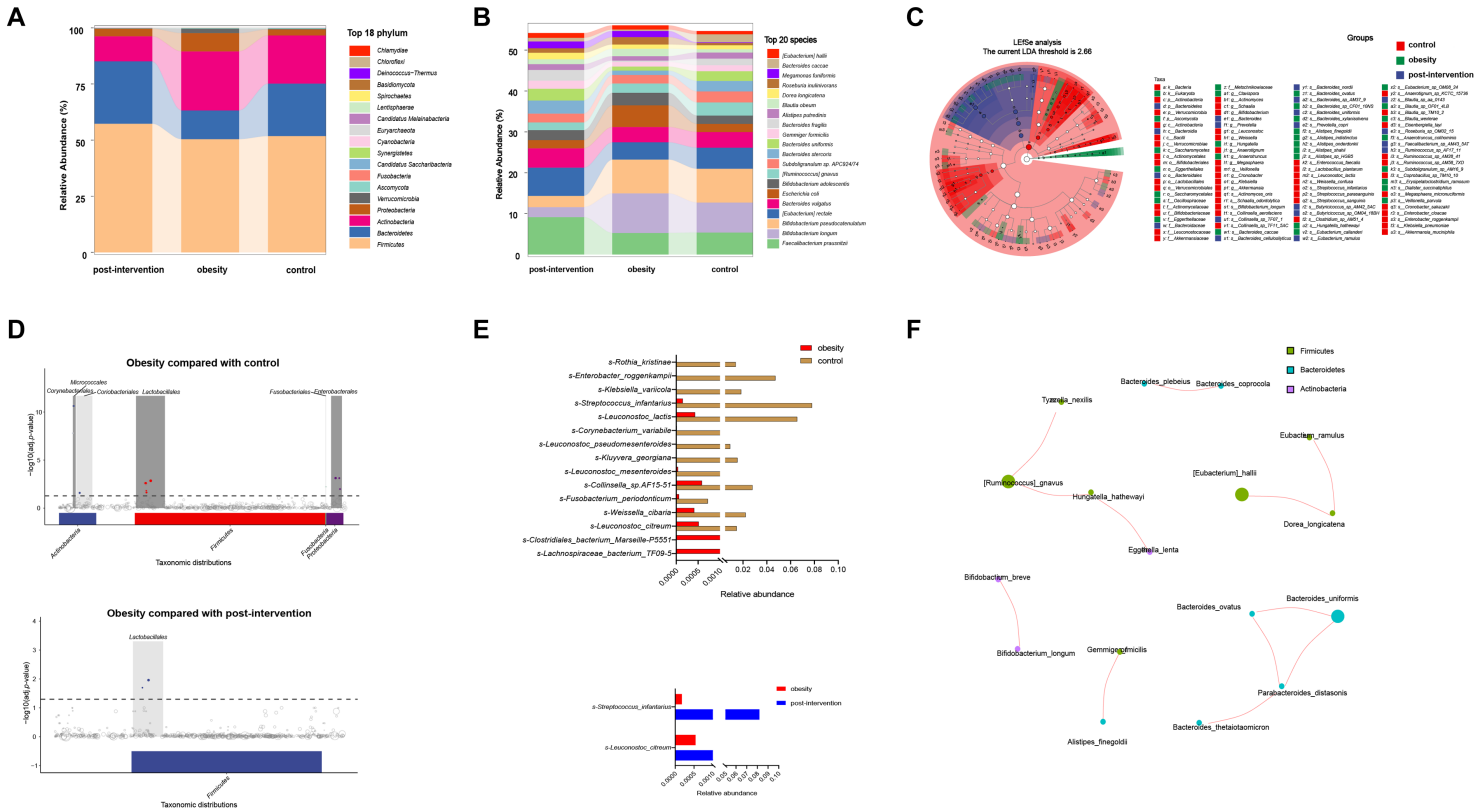
Children obesity and weight loss alter gut microbiota. Taxonomic classification at the phylum **(A)** and species **(B)** level of gut microbiota. The differences of gut microbiota among controls and children with obesity before and after weight loss intervention using LEfSe analysis (linear discriminant analysis, LDA threshold is 2.66) **(C)** and metagenomeSeq (The false discovery rate, FDA was controlled by the Benjamini-Hochberg, *adj.p* < 0.05) **(D)**. Relative abundances of fecal gut microbiota responsible for differentiation between the two groups **(E)**. The spearman correlation analysis was used to explore the co-occurrence network. The relevant networks with Spearman’s correlation, |rho| > 0.6 and *p* < 0.05 were shown **(F)**.

We obtained 47,661,359 paired-end reads on average based on shotgun metagenomic sequencing. No significant changes were observed in the Shannon, Simpson, ACE, or CHAO indices among healthy control, children with obesity before and after weight loss. A total of 2,143 species were detected, of which 1,185 overlapped among the three groups. Species accumulation curves, which were used to measure and predict the magnitude of the increase in species richness with sample size, showed that the sample size was sufficient. Additionally, the rank–abundance curve showed that the species abundance distribution in each sample was identical (Supplementary Figure S1). Differential analyses were performed from the phylum to species level using relative abundances. Wilcoxon’s test with Benjamini–Hochberg correction showed significant changes in the relative abundance of gut microbiota phyla, families, and species among the control, obesity, and post-obesity groups. Figure 2A shows the top 18 phylum in terms of overall abundance. Firmicutes (53.2%), Bacteroidetes (21.3%), and Actinobacteria (19.6%) were the major phyla identified. At the species level, the highest proportions were *s-Bifidobacterium pseudocatenulatum* (8.3%) in the control group, *s-Bifidobacterium longum* (9.7%) in the obese group, *s-Faecalibacterium prausnitzii* (9.1%) in the post-obesity group (Figure 2B). Linear discriminant analysis effect size (LEfSe) was used to compare differences in gut microbiota composition among the three groups and the current LDA threshold was set as 2.66. In the control group, higher levels of *g-Hungatella*, *s-Hungatella_hathewayi*, *s-Bacteroides_caccae*, and *s-Blautia_wexlerae* were observed. Higher levels of *p-Actinobacteriac*, c-*Actinobacteria*, and *o-Bifidobacteriales* were observed in the obesity group, whereas the post-obesity group showed higher levels of *p-Bacteroidetes*, *c-Bacteroidia*, and *o-Bacteroidales* (Figure 2C).

Next, we compared the differential relative abundances of taxa between the two groups by performing metagenomeSeq analysis. At the phylum level, Corynebacteriales, Micrococcales, Lactobacillales, Fusobacterials, and Enterobacterales were more abundant in the obese group than in the control group. Lactobacillales were significantly more abundant in the post-intervention group than in the obesity group (Figure 2D). At the species level, *s-Clostridiales_bacterium_Marseille_P5551* and *s-Clostridiales_bacterium_Marseille-P5551* levels were significantly higher in the obese group than in the control group (log_2_FC = 3.062 and 2.352, respectively). In contrast, *s-Rothia_kristinae* and *s-Enterobacter_roggenkampii* levels were significantly lower in the obese group than in the control group (logFC = −6.236 and − 4.888, respectively). After weight loss, *s-Streptococcus_infantarius* and *s-Leuconostoc_citreum* levels increased by 3.354 and 1.505 folds, respectively, compared with those before weight loss (Figure 2E). Additionally, we constructed association networks for the dominant microbial taxa in our population (Figure 2F). *S-[Eubacterium]_hallii* and *s-Dorea_longicatena* were significantly and positively correlated (*r* = 0.732, *p* < 0.01), whereas *s-Enterococcus_faecium* and *s-[Eubacterium]_rectale* were significantly negatively correlated (*r* = −0.566, *p* < 0.01).

**Figure 3:**
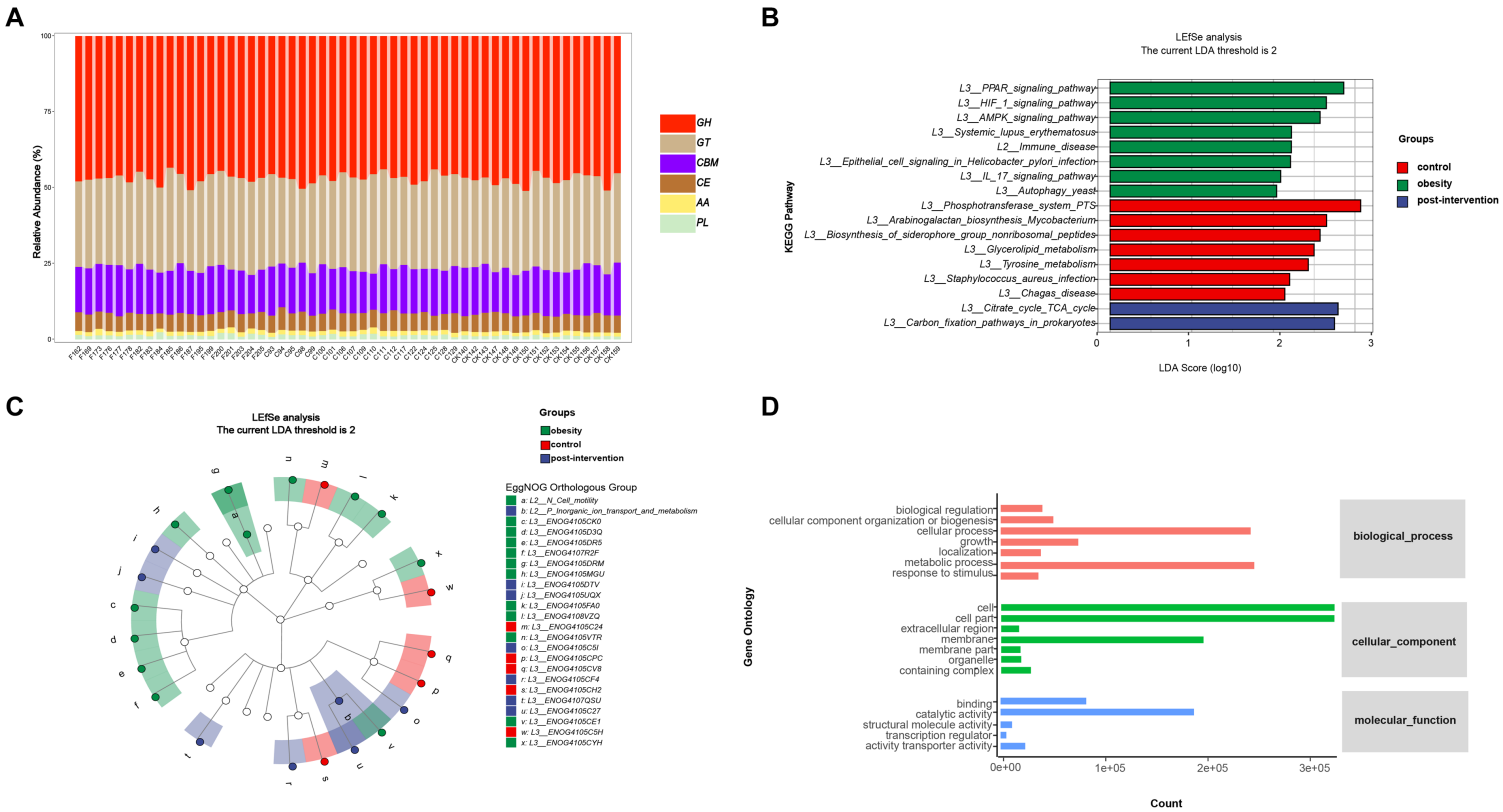
Children obesity and weight loss alter gut microbiota function. Function enrichment analysis were performed using CAZyme **(A)**, Kyoto Encyclopedia of Genes and Genomes (KEGG) **(B)**, EggNOG **(C)**, gene Ontology (GO) **(D)**, annotations. LDA threshold was 2.66.

To further examine functional differences in the gut microbiota between the obesity and control groups, CAZyme, Gene Ontology (GO), and Kyoto Encyclopedia of Genes and Genomes (KEGG) annotations were used to classify bacterial proteins. We identified 518 CAZymes for subsequent analyses (Figure 3A). Carbohydrate-binding modules and Glycoside hydrolases accounted for the highest proportion in the control group. Glycosyltransferases, auxiliary activities, carbohydrate esterases, and polysaccharide lyases accounted for the highest proportion in the obesity group. Based on the results of the KEGG analysis, the peroxisome proliferator-activated receptor and hypoxia-inducible factor 1 signaling pathways were enriched in the control group. The phosphotransferase system was enriched in the obesity group, and the citrate and tricarboxylic acid cycle (TCA) cycles were enriched in the post-obesity group (Figure 3B). The EggNOG analysis results showed that the most enriched orthologs were involved in cell wall membrane envelope biogenesis in the control group, energy production and conversion in the obesity group, and inorganic ion transport and metabolism in the post-obesity group (Figure 3C). In total, 62 GO pathways were enriched in the participants, including metabolic processes, protein-containing complexes, catalytic activities, and transporter activities (Figure 3D).

### 3.3 Serum lipidomics and correlation analyses in the obesity and control groups

**Figure 4:**
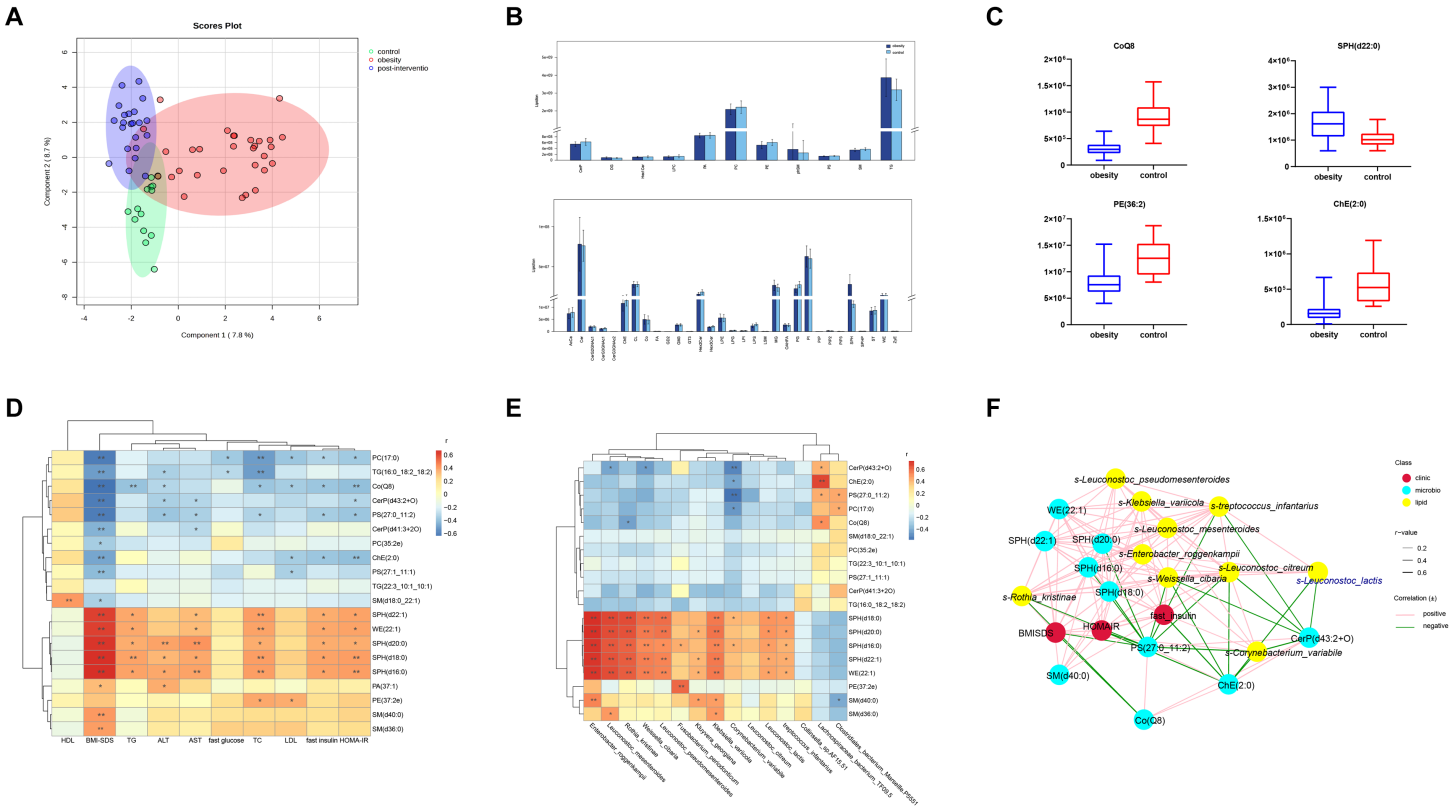
Comparisons of serum lipidomic profiles and associations of representative serum metabolites with clinical indices and gut microbial species in controls and children with obesity. **(A)** Principal component analysis (PCA) of lipidomic profile in control, children with obesity and after intervention samples. **(B)** Lipidomic profiles of controls and children with obesity. **(C)** Relative abundance of representative lipids between controls and children with obesity. Heatmap of Spearman’s correlation coefficient between the significantly different lipids and **(D)** clinical indices and **(E)** gut microbiota in controls and children with obesity. **(F)** Correlation network analysis. *p < 0.05, **p < 0.01.

Forty-two lipid classes and 4,086 lipid species were identified in our cohort. The percentage of Peak QC samples exhibiting a RSD of 30% or less constituted over 80% of the total number of Peak QC samples. The composition of lipid subclasses in each sample is presented in a circular diagram (Supplementary Figure S2). A chord diagram was used to determine the co-regulatory relationship of lipids (|r| > 0.8, *p* < 0.05). Robust associations were identified between TG levels and diacylglycerol (DG), wax esters (WE), and sphingosine (SPH) levels. The partial least squares-discriminant analysis (PLS-DA) analysis showed a significant effect of the intervention on separating children with obesity before and after weight loss from the control children (Figure 4A). The total lipid contents (TG, PI, MG, Cer, and SPH) were higher in the obesity group than in the control group (Figure 4B). At the species level, we screened 138 lipids for significant changes (VIP > 1, *p* < 0.05), of which 55 were upregulated and 83 were downregulated in the obesity group compared with those in the control group. Phosphatidic acid (PA)(37:1) was the most upregulated lipid (fold change = 12.39). Additionally, the relative abundances of SPH(d22:1) and WE(22:1) were significantly higher in the obesity group than in the control group. The top few downregulated lipids in the obesity group were SM(d18:0_22:1), PS(27:1_11:1), and ChE(2:0) (Figure 4C).

To determine the possible role of lipids in the clinic, a heatmap was used to visually represent the correlation matrix of lipids and clinical indices. The relative abundances of SPH(d22:1), WE(22:1), SPH(d18:0), SPH(d16:0), SM(d36:0), and SM(d40:0) were positively correlated with the BMI-SDS, whereas those of PC(17:0), TG(16:0_18:2_18:2), and Co(Q8) were negatively correlated. SPH(d22:1), WE(22:1), SPH(d18:0), SPH(d16:0), and PE(27:2e) levels were positively associated with fasting insulin levels and HOMA-IR. In contrast, PC(17:0), PC(17:0), PS(27:0_11:2), and ChE(2:0) levels were negatively correlated with fasting insulin levels and HOMA-IR. Among the liver enzymes, SPH(d20:0), SPH(d18:0), and SPH(d16:0) levels were negatively correlated with ALT and AST levels (Figure 4D).

Additionally, we performed a correlation analysis between significantly altered lipids and gut microbiota. The relative levels of SPH(d22:1), SPH(d20:0), SPH(d18:0), SPH(d16:0), and WE(22:1) were positively associated with the abundances of *s-Leuconostoc_mesenteroides*, *s-Enterobacter_roggenkampii*, *s-Klebsiella_variicola*, *s-Leuconostoc_lactis*, and *s-Rothia_kristinae*, respectively. PE(27:2e) levels showed a robust positive correlation with *s-Fusobacterium_periodonticum*. In contrast, SM(d40:0) levels were negatively correlated with *s-Clostridiales_bacterium_Marseille-P5551*. Additionally, CerP(d41:3 + 2O), ChE(2:0), and PS(27:0_11:2) levels were negatively correlated with *s-Collinsella_sp.AF15-51* and positively correlated with *s-Lachnospiraceae_bacterium_TF09-5* abundances (Figure 4E). Finally, a co-relationship network was conducted to seek the central traits. As shown in Figure 4F, the lipids and gut microbes were significantly associated with BMI-SDS and HOMA-IR.

### 3.4 Serum lipidomics and correlation analyses in the obesity and post-intervention groups

**Figure 5:**
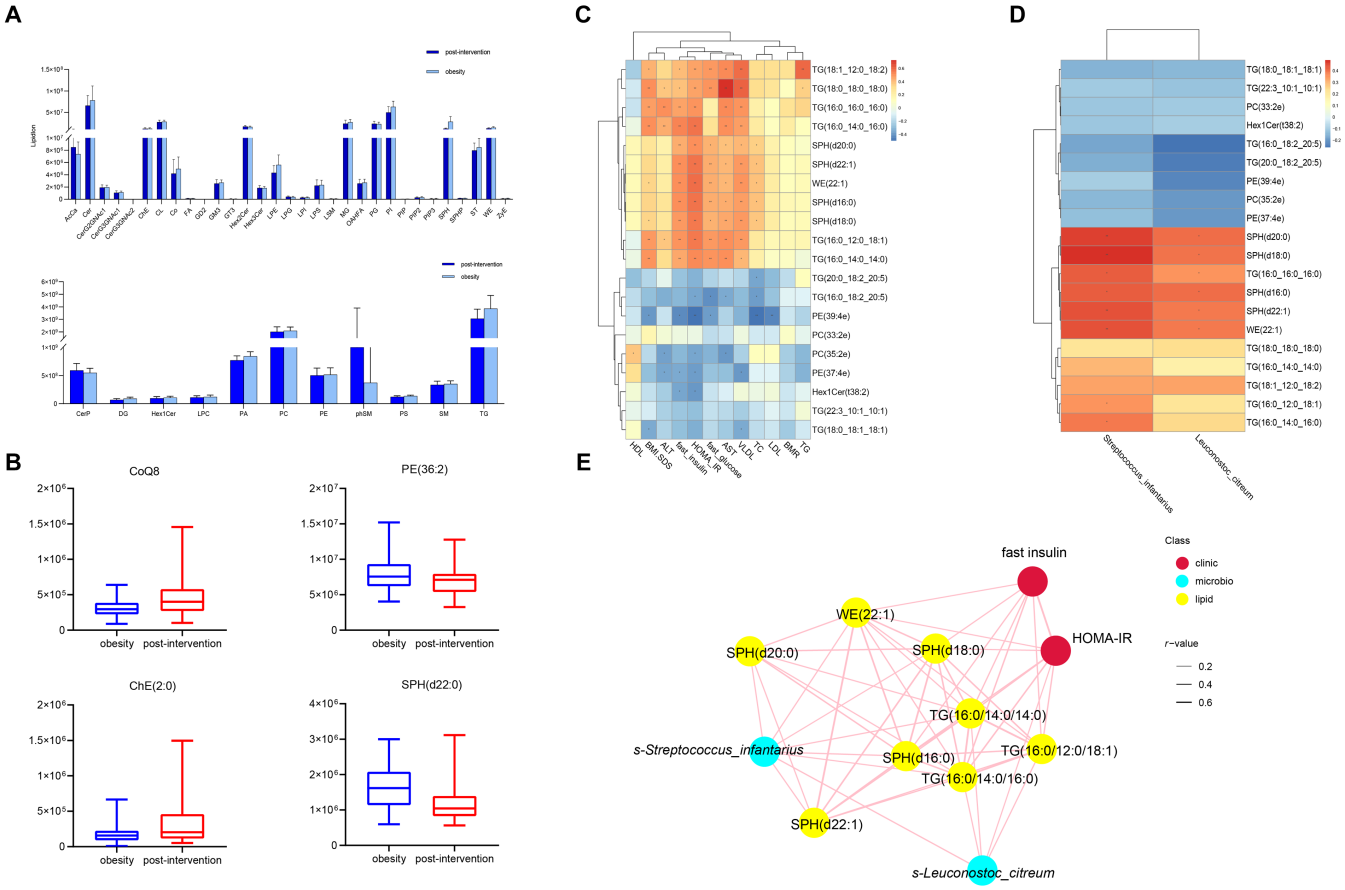
Comparisons of serum lipidomic profiles and associations of representative serum metabolites with clinical indices and gut microbial species in children with obesity before and after weight loss intervention. **(A)** Lipidomic profiles of children with obesity before and after weight loss intervention. **(B)** Relative abundance of representative lipids between children with obesity before and after weight loss intervention. Heatmap of Spearman’s correlation coefficient between the significantly different lipids and **(C)** clinical indices and **(D)** gut microbiota in children with obesity before and after weight loss intervention. **(E)** Correlation network analysis. *p < 0.05, **p < 0.01.

Lipid compositions before and after weight loss were compared in children with obesity. At the species level, we screened 177 lipids for significant changes (VIP > 1, *p* < 0.05), and 42 upregulated and 125 downregulated lipids in the post-intervention group were compared with those in the obesity group (Figure 5A). A robust association was observed between WE and SPH. The most upregulated lipid was TG(16:0_18:2_20:5) (log_2_FC = 13.71). additionally, TG(20:0_18:2_20:5) and PC(35:2e) levels were significantly higher in the post-intervention group than in the obesity group. The top few lipids downregulated in the post-intervention group were TG(16:0_14:0_14:0), SPH(d18:0), and WE(22:1). Differences observed between the children with obesity and the control indicated that the relative levels of CoQ8 and ChE(2:0) were higher after weight loss and those of SPH(d22:0) were lower than those before weight loss (Figure 5B).

Correlation analysis was performed between lipid levels and clinical parameters, with significant changes before and after weight loss. The relative levels of SPH(d20:0), SPH(d22:1), WE(22:1), SPH(d18:0), SPH(d16:0), and SM(d40:0) were positively correlated with BMI-SDS, whereas those of TG(16:0_18:2_20:5), PE(39:4e), and PE(37:4e) were negatively correlated. SPH(d20:0), SPH(d22:1), WE(22:1), SPH(d18:0), SPH(d16:0), and SM(d40:0) levels were positively correlated with fasting insulin levels and HOMA-IR. In contrast, Hex1Cer(t38:2), PE(37:4e), and PE(39:4e) levels were negatively correlated with fasting insulin levels and HOMA-IR (Figure 5C). The altered microbiota and lipids were correlated with each other. *S-Streptococcus_infantariu* and *s-Leuconostoc_citreum* abundances were significantly and positively correlated with SPH(d20:0), SPH(d22:1), WE(22:1), SPH(d18:0), SPH(d16:0), and TG(16:0_16:0_16:0) levels (Figure 5D). Finally, the co-relationship network identified that significantly changed lipids and gut microbes were associated with HOMA-IR.

## 4 Discussion

The gut microbiota and lipids modulate obesity. However, limited information is available regarding their effects on children with obesity. In this study, we examined changes and correlations in gut microbiota and serum lipid metabolism in children with obesity before and after weight loss and healthy controls. The principal novel findings of this research indicated significant variations in the lipid compositions of fecal and blood samples across the three studied groups. We identified specific lipid and gut microbiota markers that exhibited a strong correlation with childhood obesity. Furthermore, notable alterations in gut microbiota functions were detected in children with obesity following weight loss interventions.

Dyslipidemia and pathoglycemia are common complications of childhood obesity. Our findings showed that the obesity group showed significantly higher fasting glucose, HOMA-IR, TC, LDL, ALT, and AST levels than did the control group, and these levels significantly improved after weight loss with the life intervention. Treatment options for childhood obesity are limited, and no drugs are currently available to treat the condition. To prevent obesity, it is necessary to ensure healthy linear growth in children while restricting their calorie intake. Most children in the obese group managed to lose weight with supervision and rewards from both investigators and parents. This indicated that the life intervention exerted a substantial effect on weight loss in children with obesity. As the duration of the intervention increased, long-term supervision could not be effectively guaranteed, and four children dropped out. This finding indicates that lifestyle interventions have some limitations. It is imperative to investigate alternative interventions such as altering the gut microbiota and identifying possible therapeutic targets.

The metagenomics analysis revealed no significant differences in the diversity of gut microbiota between children with obesity and the controls. Firmicutes and Bacteroidetes abundances significantly decreased in children with obesity and increased after weight loss, which was consistent with previous results (23). In the present study, we found that *s-Lachnospiraceae_bacterium_TF09-5* and *s-Clostridiales_bacterium_Marseille-P5551* levels increased significantly in children with obesity. Zhu et al. (24). showed that *f-Lachnospiracea* was enriched in obesity-sensitive mice. Additionally, we found increased *s-Clostridiales_bacterium_Marseille-P5551* abundance in children with obesity. We identified 13 probiotics that were significantly decreased in these children. The top decreased species was *s-Rothia_kristinae*. Liver steatosis and inflammation improved significantly when an alcoholic fatty liver mouse model was supplemented with Rothia (25). A study on the effects of *s-Leuconostoc_pseudomesenteroides* showed its role in reducing body weight and improving dihydroceramide levels in high-fat diet-induced obese mice (26). Additionally, *s-Kluyvera_georgiana* was inversely associated with 25-hydroxyvitamin D levels in women with obesity suffering from polycystic ovary syndrome (27). These findings indicated that gut microbiota was substantially altered in children with obesity. After the lifestyle intervention, *s-Streptococcus_infantarius* and *s-Leuconostoc_citreum* abundances increased significantly. These two microbiotas are closely related to milk fermentation, indicating that changes in the dietary structure during the process of weight loss in children affect the gut microbiota (28). We analyzed differences in microbiota functions between different groups. Glycosyltransferase and phosphotransferase system pathways were enriched in the obesity group, suggesting that gut microbiota also plays a role in host modification. Glycosylation and phosphorylation modifications in individuals with obesity affect protein functions such as glucose transport, lipoprotein binding, and inflammation (29). The changes in carbon fixation and the TCA cycle after weight loss indicated that gut microbiota also played a role in the process of weight loss.

Lipid species play critical roles in cellular structure, energy metabolism, and cell signaling. Lipid dysregulation is closely associated with obesity progression. As shown by PLS-DA, lipidomics could clearly distinguish between the controls, children with obesity, and children with obesity after weight loss. The total abundance of diglycerides and phosphatidylinositol was significantly higher in children with obesity and decreased after weight loss, which is consistent with the results in adults with obesity (30). However, several studies have reported that the levels of several PI species are lower in children with obesity than in controls (31–33). Phosphatidylinositol (PI)(36:3), PI(38:1), PI(39:6), PI(40:4), and PI(44:6e) levels were lower in children with obesity at the subspecies level. Phosphatidylethanolamine (PE)(37:2e) was associated with TC and LDL levels. In a study on adults with obesity, increased phosphatidylethanolamine levels increased susceptibility to nonalcoholic fatty liver disease (NAFLD), inhibited hepatocyte proliferation, and induced inflammation (34). Draijer et al. (35) identified PE as a potential biomarker of pediatric NAFLD. However, PE(37:4e) and PE(39:4e) were negatively related to ALT levels after the intervention, proving that chain length and saturation affect PE function differently in children with obesity. LPC is produced through the cleavage of phosphatidylcholine and plays a role in cholesterol biosynthesis and fatty acid oxidation (36). Research conducted by Sharma et al. (37) indicates that elevated plasma LPC levels in children with obesity correlate with BMI, a finding that aligns with our own results.

Sphingolipids are the second most abundant membrane lipids. The relative abundances of sphingolipids and ceramides were significantly higher in the obesity group than in the control group. Sphingolipid and ceramide levels increased significantly after weight loss. Moderate-intensity exercise without dietary intervention also induces significant sphingolipid reduction (38). Most SPHs and ceramides were positively correlated with metabolic parameters in our cohort. Hellmuth et al. (20) found that SM(32:2) was closely related to the BMI z-score. Sphingosine-1-phosphate affects insulin signaling via sphingosine-1-phosphate receptors (39). Ceramide content increases in the presence of excess fatty acids and affects insulin-stimulated Akt activation (40). SPHs positively correlated with *s-Enterobacter_roggenkampii*. A recent study demonstrated that sphingolipids produced by gut bacteria could enter host metabolic pathways and affect ceramide levels (41). Given that SPHs were associated with most of the significantly altered microbiota, it is likely that the changes in SPHs were a consequence, rather than a cause, of the changes in gut microbiota. There are a few reports on hexosylceramide levels in children with obesity. Decreased hexosylceramide levels have been observed in children with obesity. In addition, Her1Cer(t38:2) levels were negatively associated with HOMA-IR. A previous study reported that hexosylceramide levels were inversely associated with dysmetabolic biomarkers (42).

We found that CoQ8 levels were negatively correlated with most obesity-related indicators, reflecting reduced coenzyme metabolism in children with obesity. CoQ8 plays a critical role in increasing and streamlining coenzyme Q production (43). CoQ supplementation rescued ceramide-associated IR (44). A positive correlation was observed between CoQ8 levels and *s-Lachnospiraceae_bacterium_TF09-5* abundance. Coenzyme Q biosynthesis is also observed in bacteria (45). The mechanism of action of CoQ8 in obesity requires further exploration.

In the joint analysis of lipids and gut microbiota, ChE(2:0) levels were positively correlated with *s-Lachnospiraceae_bacterium_TF09-5* but negatively correlated with *s-Corynebacterium_*var*iabile*. A recent study showed that cholesteryl esters synthesized by hepatocytes and enterocytes affected β-oxidation and glucose metabolism (46). Taken together with the significant positive correlation between ChE levels and HOMA-IR, it is reasonable to hypothesize that gut microbiota may affect glucose metabolism via ChE.

To the best of our knowledge, the current investigation represents the inaugural effort to integrate untargeted lipidomics and gut metagenomics in order to evaluate the lipid and gut microbiota profiles among normal-weight children, children with obesity, and children with obesity following weight loss. Previous research has established a connection between gut microbiota and lipid profiles in children with obesity. Nonetheless, this study is not without its limitations. Firstly, the research was conducted with a relatively small sample size within a pediatric clinic, which may introduce selection bias. Secondly, the simultaneous collection of serum and stool samples precludes the establishment of a causal relationship between lipids and gut microbiota. Thirdly, variations in sociodemographic factors and dietary habits may exert some influence on gut microbiota composition. Despite these limitations, the present study primarily focuses on the effects of dietary energy restriction and lifestyle modifications on childhood obesity. Consequently, there is a pressing need for a multi-center study to enhance the sample size. Furthermore, the implementation of questionnaires addressing socioeconomic factors and diverse dietary patterns is recommended to further investigate the impact of these variables on childhood obesity and weight loss through gut microbiota in future research endeavors.

In conclusion, the present study has identified a relationship between altered gut microbiota and serum lipid concentrations in children with obesity, along with associated clinical markers. The findings contribute to our understanding of the mechanistic relationship between modified gut microbiota and childhood obesity, as well as weight reduction, indicating that the gut microbiome may serve as a viable target for interventions aimed at reducing weight in pediatric populations.

## Data Availability

The datasets presented in this study can be found in online repositories. The names of the repository/repositories and accession number(s) can be found at: https://www.ncbi.nlm.nih.gov/, PRJNA1090975.

## Supplementary material

SUPPLEMENTARY FIGURE S1 Species diversity analysis of the metagenome of gut microbiota. **(A)** Alpha diversity in controls, children with obesity and after intervention represented by Shannon, Simpson, ACE, or CHAO indices using pairwise Wilcoxon rank sum test (*p* > 0.05 for measured index). **(B)** Venn diagram of three groups of gut microbiota species were obtained after identification. **(C)** The species accumulation curves of all subjects. **(D)** The rank–abundance curves of all subjects.

SUPPLEMENTARY FIGURE S2 Categorical composition of serum lipidomics. The subclass of lipids number **(A)** of all subjects and proportion of controls **(B)**, children with obesity **(C)** and after weight loss intervention **(D)** were shown.
