# Supplementary figures and images for: Integrative metagenomic and lipidomic analyses reveal alterations in children with obesity and after lifestyle intervention
Source: Front Nutr. 2024 Sep 10;11:1423724. doi: 10.3389/fnut.2024.1423724 (PMC11420138; doi:10.3389/fnut.2024.1423724)

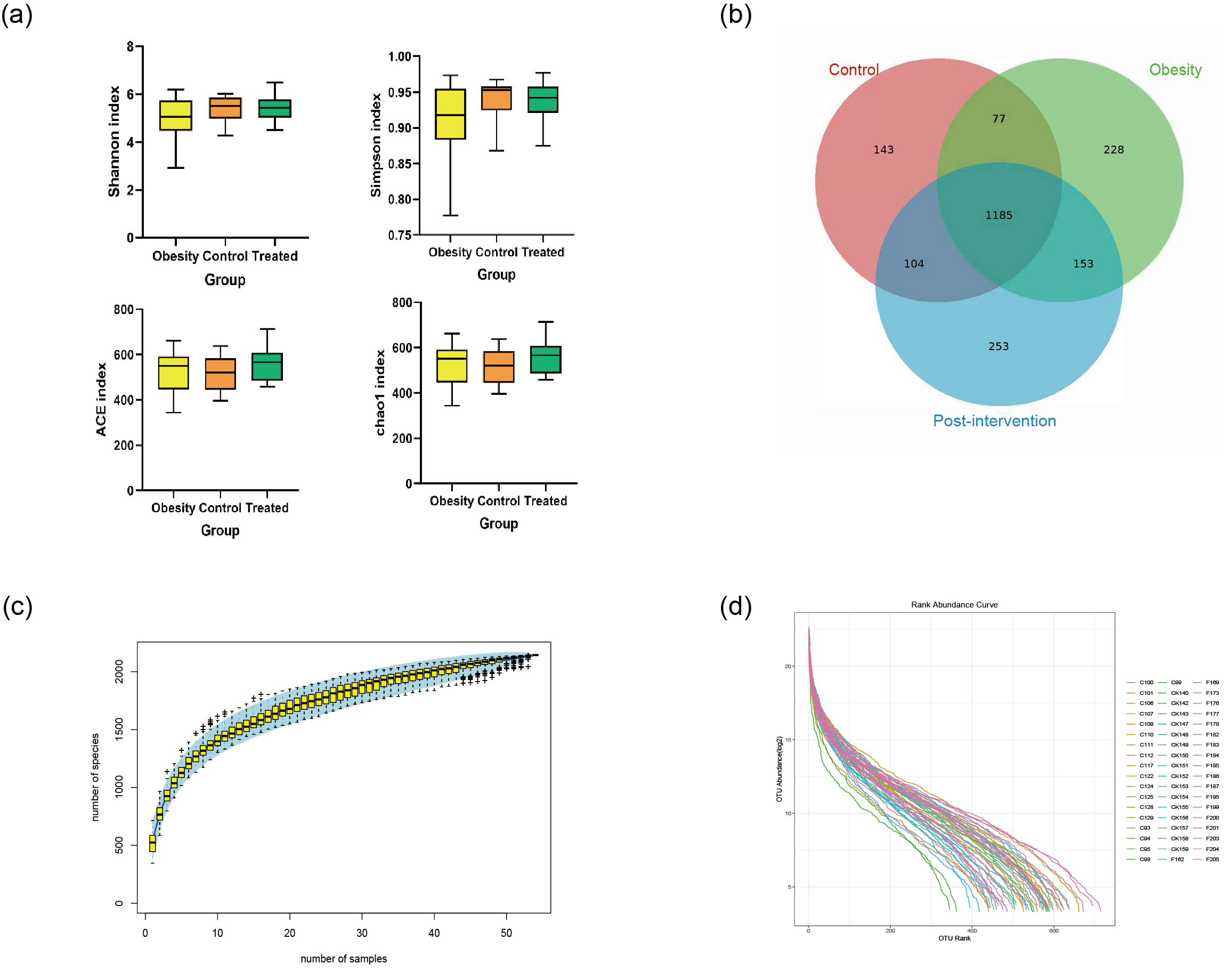

Supplement: SUPPLEMENTARY FIGURE S1 — Species diversity analysis of the metagenome of gut microbiota. (A) Alpha diversity in controls, children with obesity and after intervention represented by Shannon, Simpson, ACE, or CHAO indices using pairwise Wilcoxon rank sum test (p > 0.05 for measured index). (B) Venn diagram of three groups of gut microbiota species were obtained after identification. (C) The species accumulation curves of all subjects. (D) The rank–abundance curves of all subjects. [file Image_1.jpeg]

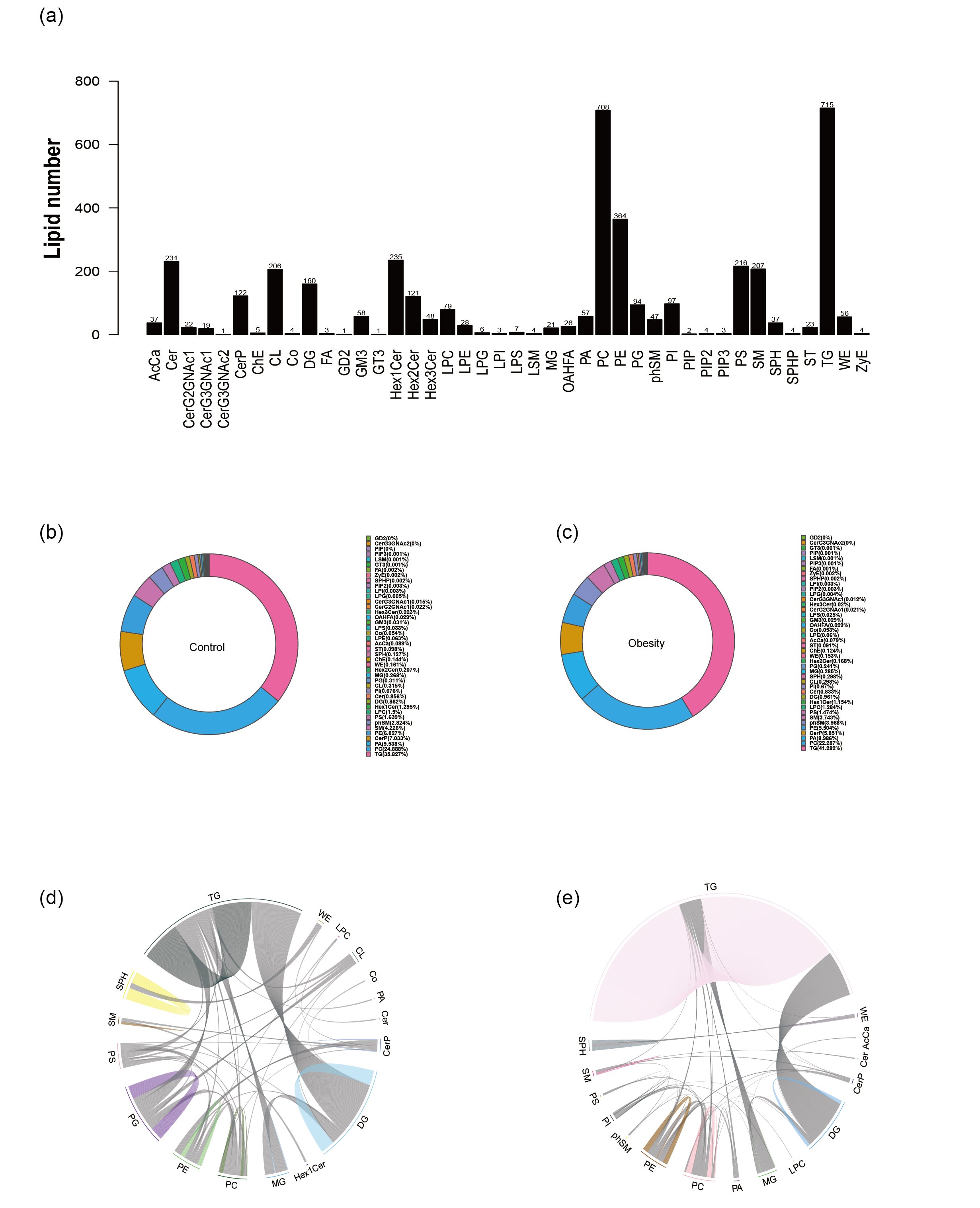

Supplement: SUPPLEMENTARY FIGURE S2 — Categorical composition of serum lipidomics. The subclass of lipids number (A) of all subjects and proportion of controls (B), children with obesity (C) and after weight loss intervention (D) were shown. [file Image_2.jpeg]
